# MED15/382: Co-operative Interface in Medical Education on the WWW

**DOI:** 10.2196/jmir.1.suppl1.e58

**Published:** 1999-09-19

**Authors:** C Courtin, P Le Beux

**Affiliations:** ^1^PROTIMES - Faculté de Médecine, RennesFrance

**Keywords:** Internet, User-Computer Interface, Medical Education, Computer Supported Co-operative Work

## Abstract

Hospitals deal with a huge volume of medical information shared by multidisciplinary staff. The management and circulation of this information require massive computerization. This medical computerization, associated with the Internet technological progress, has given birth to many software applications in the educational field. In the context of medical virtual university, the World Wide Web intrinsic interactive and asynchronous functionality has to be extended to become co-operative and in real-time. In the context of our virtual medical university in Rennes, we have worked on a system, called CSCWeb, allowing implementation of synchronous co-operative functionality through multi-platform graphical interfaces on the Web. This project is motivated by the use of existing computer-based equipment in most of medical schools. The CSCWeb system is designed to be easily upgraded by Java programmers. It allows to re-use generic co-operative graphical tools, such as textual chat, and to develop specific graphical tools well-suited to application fields. In the CSCWeb system, the participants of a co-operative session give automatic individual access to the multimedia medical information. This distributed access avoids the bottleneck generally caused by multimedia data volume in centralized systems. The system handles only internal and external data. The former are commands which allow to play remotely the same sequence of graphical events of the local user's interface. The latter are additional medical information, such as comments, definition of region of interest on a X-ray, etc. These internal and external data are textual (comments, co-ordinates, etc.), and represent a small volume of data to transfer between participants. Furthermore, these commands are only dependent on graphical objects (button, frame, etc) whatever their use is (color selector, etc). Therefore, the use of commands allows easy extension of co-operative functionality to any kind of data, such as video, without increasing the charge of the CSCWeb mechanism. Only individual access to the new type of data will be affected by its volume according to the network bandwidth. Our first purpose was to check the feasibility of our strategic choices. To date, we have implemented an educational prototype including a group management interface which is a built-in co-operative graphical tool, and a whiteboard which is a generic one. The resulting system has been evaluated, on intranet in the Laboratory of Medical Computing in Rennes (France), in terms of implementation and working performance. This evaluation is encouraging to extend the CSCWeb system for co-diagnosis in medical practice. As it is platform independent, the CSCWeb system is well-adapted to almost all medical units' computer-based equipment. Furthermore, the great flexibility of the Java programming language in user interface design contributes to fit final users' requirements.

